# Non-invasive goal-directed fluid therapy with the pleth variability index (PVI): a systematic review and meta-analysis

**DOI:** 10.1007/s10877-025-01334-7

**Published:** 2025-08-08

**Authors:** Vitor Alves Felippe, Roberta Codeceira, Maria Irigaray, Maria Sckaff, Bruno Wegner, Tatiana Nascimento, Carlos Darcy, Lucas Dutra, Bruno Santiago, Julia Buchmann, Marcos Adriano Lessa

**Affiliations:** 1 Department of Anesthesiology, Brazilian National Cancer Institute, Rio de Janeiro, Brazil; 2 School of Medicine, Federal University of Rio de Janeiro, Rio de Janeiro, Brazil; 3 School of Medicine, University of the Region of Joinville (UNIVILLE), Joinville, Brazil; 4 School of Medicine, Johns Hopkins University, Baltimore, USA; 5 School of Medicine, Federal University of Rio Grande do Sul, Porto Alegre, Brazil; 6 Department of Anesthesia, University of Iowa, Iowa City, USA; 7 Translational Medicine, Paulista School of Medicine (EPM-UNIFESP), São Paulo, Brazil; 8 Department of Anesthesiology, Ipanema Federal Hospital, Rio de Janeiro, Brazil; 9 IDOMED, Institute for Medical Development, Rio de Janeiro, Brazil; 10 Department of Surgery, Anesthesia Division, State University of Rio de Janeiro (UERJ), Rio de Janeiro, Brazil; 11 Department of Anesthesia, Carver College of Medicine, University of Iowa, 200 Hawkins Dr 6413 JCP, Iowa City, IA 52242 USA

**Keywords:** Pleth Variability Index (PVI), Goal-directed fluid therapy, Perioperative fluid management, Systematic review, Meta-analysis

## Abstract

Optimal intraoperative fluid management is essential to improve surgical outcomes and reduce complications. The Pleth Variability Index (PVI), a dynamic and non-invasive indicator of fluid responsiveness, has been proposed as a tool for goal-directed fluid management. This systematic review and meta-analysis aimed to evaluate the effectiveness of PVI-guided fluid therapy compared to conventional fluid management (CFM) in non-cardiac surgeries. A comprehensive search of PubMed, Embase, and Cochrane databases up to January 2024 identified eligible studies. Primary outcomes included total intraoperative fluid volume and crystalloid administration. Secondary outcomes included hemodynamic parameters, renal function markers, acid-base balance, and hospital length of stay (LOS). Random-effects models were applied, and subgroup and sensitivity analyses were performed. Nine studies comprising 1,105 patients were included. Compared to conventional fluid management, PVI-guided therapy significantly reduced total fluid volume (mean difference [MD] − 761.23 mL; 95% CI − 1267.42 to − 255.03) and crystalloid administration (MD − 655.05 mL; 95% CI − 1096.48 to − 213.62), without significant differences in colloid use, urine output, norepinephrine requirement, arterial pressure, acid-base balance, or LOS. Subgroup analysis of abdominal surgeries confirmed the observed reduction in fluid volumes. PVI-guided fluid management allows for a more restrictive and individualized approach without compromising hemodynamic or metabolic stability. While the heterogeneity across studies limits generalizability, these findings support the clinical value of PVI as a non-invasive tool for perioperative fluid optimization, especially in settings where invasive monitoring is not feasible. Further trials are needed to evaluate its impact on long-term outcomes.

**Supplementary Information:**

The online version contains supplementary material available at 10.1007/s10877-025-01334-7.

## Introduction

Modern anesthesia extends beyond inducing unconsciousness; It incorporate precision medicine strategies to optimize perioperative hemodynamics and improve patient outcomes. Among these, Precise intraoperative fluid management plays a pivotal role, as both hypovolemia and hypervolemia can contribute to significant complications during the perioperative period [1, 2, 3, 4, 5], increasing morbidity and mortality [6], hospital stays [7, 8], and healthcare costs [9]. Achieving an optimal fluid balance is therefore a cornerstone of perioperative care, particularly in high-risk surgical populations.

Conventional fluid management (CFM) relies on static parameters such as heart rate, blood pressure, and urine output. However, these measures have limited predictive value for fluid responsiveness, leading to inaccurate intravascular volume assessment and suboptimal fluid administration. To address this challenge, dynamic parameters such as Stroke Volume Variation (SVV), Pulse Pressure Variation (PPV), and the Pleth Variability Index (PVI) have been introduced to guide individualized fluid therapy strategies [10]. PVI, derived from the respiratory variations in the pulse oximeter waveform, offers a non-invasive and continuous assessment of fluid responsiveness, making it an attractive alternative to more invasive monitoring methods [11].

Although prior studies have explored PVI’s predictive accuracy, the evidence regarding its effectiveness in guiding intraoperative fluid therapy remains fragmented. Some reports suggest that PVI-guided goal-directed fluid management (GDFM) can reduce fluid administration while maintaining hemodynamic stability, but findings have been inconsistent across different surgical settings [12, 13, 14]., Furthermore, questions remain regarding the impact of PVI-guided therapy on postoperative recovery and whether it should complement or replace existing hemodynamic monitoring techniques [15].

This systematic review and meta-analysis aim to consolidate current evidence on the efficacy of PVI-guided fluid management in non-cardiac surgeries. Specifically, we assess whether PVI-based GDFM optimizes intraoperative fluid administration without compromising hemodynamic stability, acid-base balance, or postoperative outcomes. By addressing the limitations of previous studies, we seek to clarify PVI’s role in perioperative medicine and identify areas for future research.

## Methods

### Study design and registration

This systematic review and meta-analysis were conducted following the Preferred Reporting Items for Systematic Reviews and Meta-Analyses (PRISMA) [16] and the Cochrane Collaboration Handbook for Systematic Review of Interventions [17]. The study protocol was registered in the International Prospective Register of Systematic Reviews under the registration number CRD42025649617.

### Eligibility criteria

Studies were included if they met the following criteria: 1- randomized controlled trials (RCTs), 2- comparing PVI-guided goal-directed fluid management (GDFM) to CFM, 3- conducted in patients undergoing non-cardiac surgeries, and 4- reporting at least one relevant perioperative outcome.

We excluded studies that 1- involved duplicate or overlapping populations, 2- focused on pediatric or obstetric patients, or 3- did not specifically use PVI as a fluid responsiveness parameter. PVI-GDFM was defined as intraoperative fluid administration based on dynamic PVI monitoring, whereas CFM relied on conventional static parameters such as heart rate, blood pressure, and urine output.

### Search strategy and data extraction

A systematic search was conducted in PubMed, Embase, and the Cochrane Central Register of Controlled Trials (CENTRAL) from inception to January 2024 using the following search terms: “Pleth Variability Index” OR “PVI” OR “Pulse Oximeter Variability” OR “Plethysmographic Variability” OR “Non-Invasive Monitoring”. The complete search strategy is detailed in Supplementary Methods in Supplementary Data.

Additionally, reference lists of all included studies, as well as previous systematic reviews and meta-analyses, were manually screened to identify any additional relevant studies. Two independent reviewers (V.F. and R.C.) extracted data using standardized spreadsheets. Extracted data included study characteristics, number and demographics of participants in each group, type of surgical procedure, intraoperative fluid management strategy, and reported clinical outcomes.

### Outcomes

This meta-analysis primarily outcome was intraoperative fluid balance, with a specific focus on total fluid, crystalloid and colloids infusion. Secondary outcomes included norepinephrine requirement, mean arterial pressure (MAP), urine output, 24-hour creatinine, arterial pH, bicarbonate, lactate levels, blood loss, and hospital length of stay (LOS).

### Risk of bias assessment

Two authors (V.F. and B.W.) independently assessed the risk of bias. Disagreements were resolved with a third author. The Cochrane Risk of Bias-2 (RoB-2) tool was used to evaluate the risk of bias in randomized trials. RoB-2 evaluates five domains: randomization process, deviations from intended interventions, missing outcome data, measurement of the outcome, and selection of the reported result [18].

### Publication bias assessment

Publication bias was evaluated through visual inspection of funnel plots and analysis of control lines. Due to the limited number of included studies, no formal quantitative assessment of small-study effects or publication bias was conducted.

#### Sensitivity analyses

To explore potential sources of variability and address heterogeneity, we conducted subgroup analyses, categorizing studies based on key characteristics such as abdominal surgery. Additionally, we performed sensitivity analyses to assess the robustness of our findings. Specifically, we applied a leave-one-out (LOO) approach [19] for total fluid infused and crystalloids infused, sequentially removing each study one at a time and reanalyzing the data. This method allowed us to evaluate the influence of individual studies on the pooled results and ensure the stability of our findings.

### Statistical analysis

We pooled risk ratios (RR) and mean differences (MD) with 95% confidence intervals (CI) for categorical and continuous outcomes, respectively. To account for heterogeneity in methodology and demographics across studies, we applied a random-effects model [20]. We assessed heterogeneity with I² statistics and Cochrane Q test; p-values < 0.10 and I² >25% were considered significant for heterogeneity. Statistical analyses were performed using R statistical software, version 4.2.3 (R Foundation for Statistical Computing) [21].

## Results

### Study selection and characteristics

**Fig. 1:**
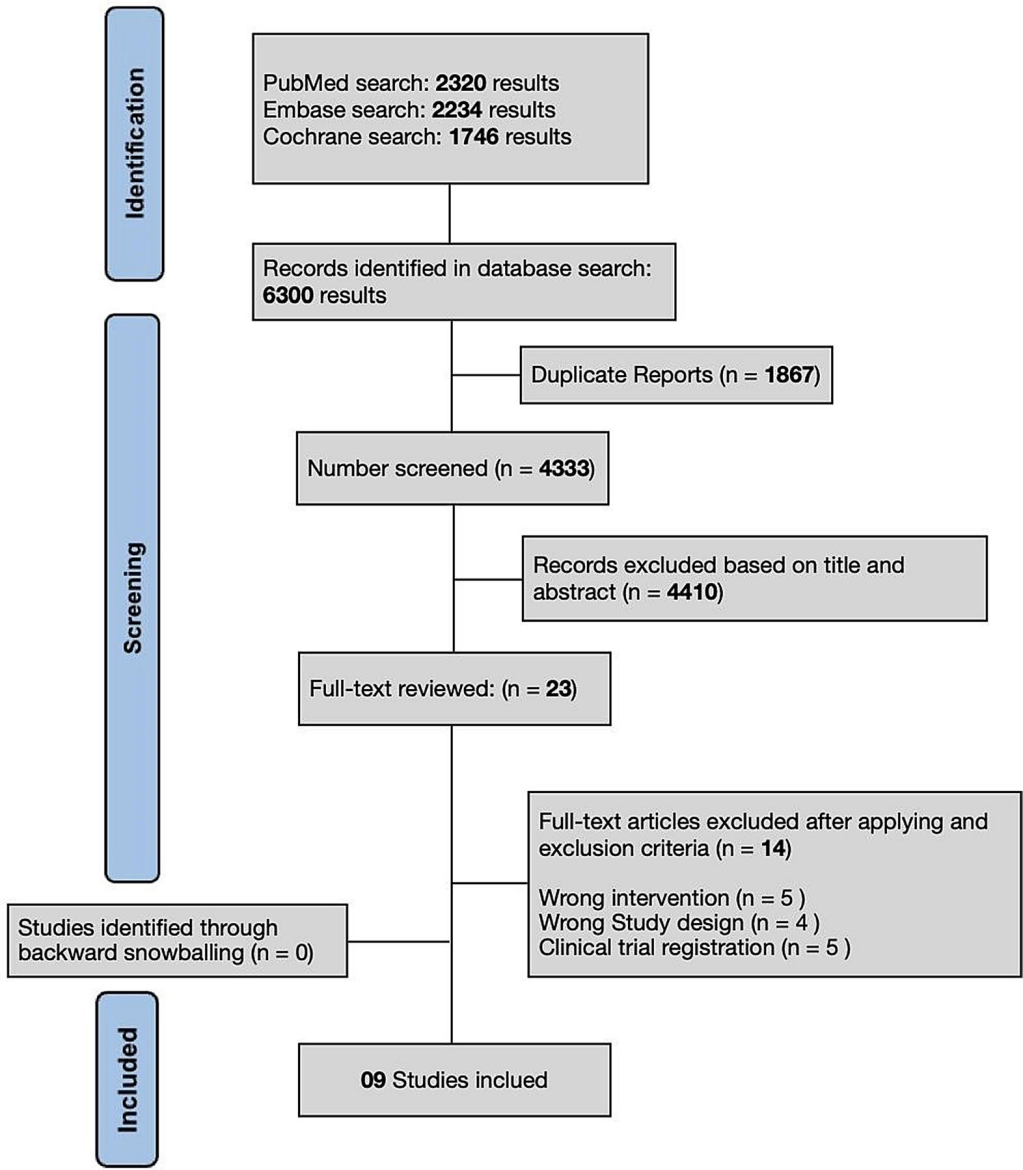
PRISMA flow diagram detailing the study selection process, including identification, screening, and inclusion of studies in the meta-analysis

The initial database search conducted on November 8, 2024, identified 4,433 records. After removing duplicates and applying the eligibility criteria, 23 studies were selected for full-text review (Fig. 1). Of these, 9 studies met the inclusion criteria and were included in this systematic review and meta-analysis [15, 22, 23, 24, 25, 26, 27, 28, 29]. The primary reasons for exclusion were: (1) Studies not specifically evaluating PVI- GDFM; (2) Not meet the predefined methodological criteria.

A total of 1,105 patients were included across the selected studies, with 551 patients receiving PVI-GDFM and 554 receiving CFM. The overall mean patient age was 54.4 years, with similar baseline across studies (Table 1). The surgical procedures included abdominal, orthopedic, gynecological, and major general surgeries, and enrollment periods spanning studies published between 2010 and 2023 were generally comparable, as summarized in (Table 1).

**Table 1:** Baseline characteristics of the included studies

Study	Patients (*n*)	Age (M ± SD) PVI/CFM	Sex % Fem	BMI (M ± SD) PVI/CFM	ASA (I/II/III) PVI/CFM	Type of surgery	PVI Threshold for Intervention
Abdelhamid 2023	66	40.4 ± 10.9 / 41.9 ± 9.8	42.4%	27.4 ± 3.5 / 26.4 ± 2.5	29/4/0 / 27/6/0	Elective lumbar spine procedures under general anesthesia in the prone position	> 13% for > 5 min
Cesur 2019	70	58.7 ± 14.4 / 62.3 ± 10.5	41.4%	25.5 ± 4.9 / 26.8 ± 4.2	22/13/0 / 19/16/0	Elective colorectal surgery	> 13% for > 5 min
Demirel 2017	60	36.3 ± 10.8 / 40.1 ± 11.9	61.7%	43.1 ± 3.3 / 43.6 ± 4.5	0/15/15 / 0/12/18	Laparoscopic Roux-en-Y Gastric Bypass Surgery in Morbidly Obese Patients	> 14% for 5 min
Fischer 2020	438	65.0 ± 10.0 / 66.0 ± 10.0	36.8%	29.0 ± 6.0 / 30.0 ± 6.0	36/149/28 / 30/152/39	Adult patients having elective orthopedic surgery	> 13%
Forget 2010	82	59.0 ± 14.0 / 61.0 ± 12.0	39.0%	NA	0/22/19 / 0/22/19	Major abdominal surgery under general anesthesia	> 13% for ≥ 5 min
Hokenek 2022	78	48.7 ± 5.4 / 48.7 ± 6.1	NA	30.9 ± 5.6 / 30.0 ± 4.7	All patients ASA I/II/III	Elective total abdominal hysterectomy and bilateral salpingo-oophorectomy under general anesthesia	> 13% (when MAP ≤ 65 mmHg and/or > 20% decrease in MAP)
Wang 2023	211	70.7 ± 5.4 / 70.7 ± 5.2	31.3%	22.4 ± 3.0 / 22.9 ± 3.2	12/80/15 / 7/79/18	Major gastrointestinal surgical in elderly patients	> 13% (when MAP ≥ 65 mmHg) for 5 min
Yilmaz 2022	64	53.0 ± 6.0 / 51.0 ± 8.0	NA	31.5 ± 5.4 / 31.9 ± 5.8	3/21/5 / 2/29/3	Elective laparoscopic total hysterectomy	> 13%
Yu 2014	30	58.0 ± 5.0 / 59.0 ± 6.0	43.3%	NA	All patients ASA I/II	Elective abdominal surgery under combined general and epidural anesthesia	> 13% over 5 min

n, number of patients; PVI, pulse variability index; M, mean; SD, standard deviation; CFM, conventional fluid management; ASA, American Society of Anesthesiologists; BMI, body mass index (kg/m^2^); MAP, mean arterial pressure; min, minutes; Fem, female

### Primary outcomes: fluid administration

#### Total fluid infused

**Fig. 2:**
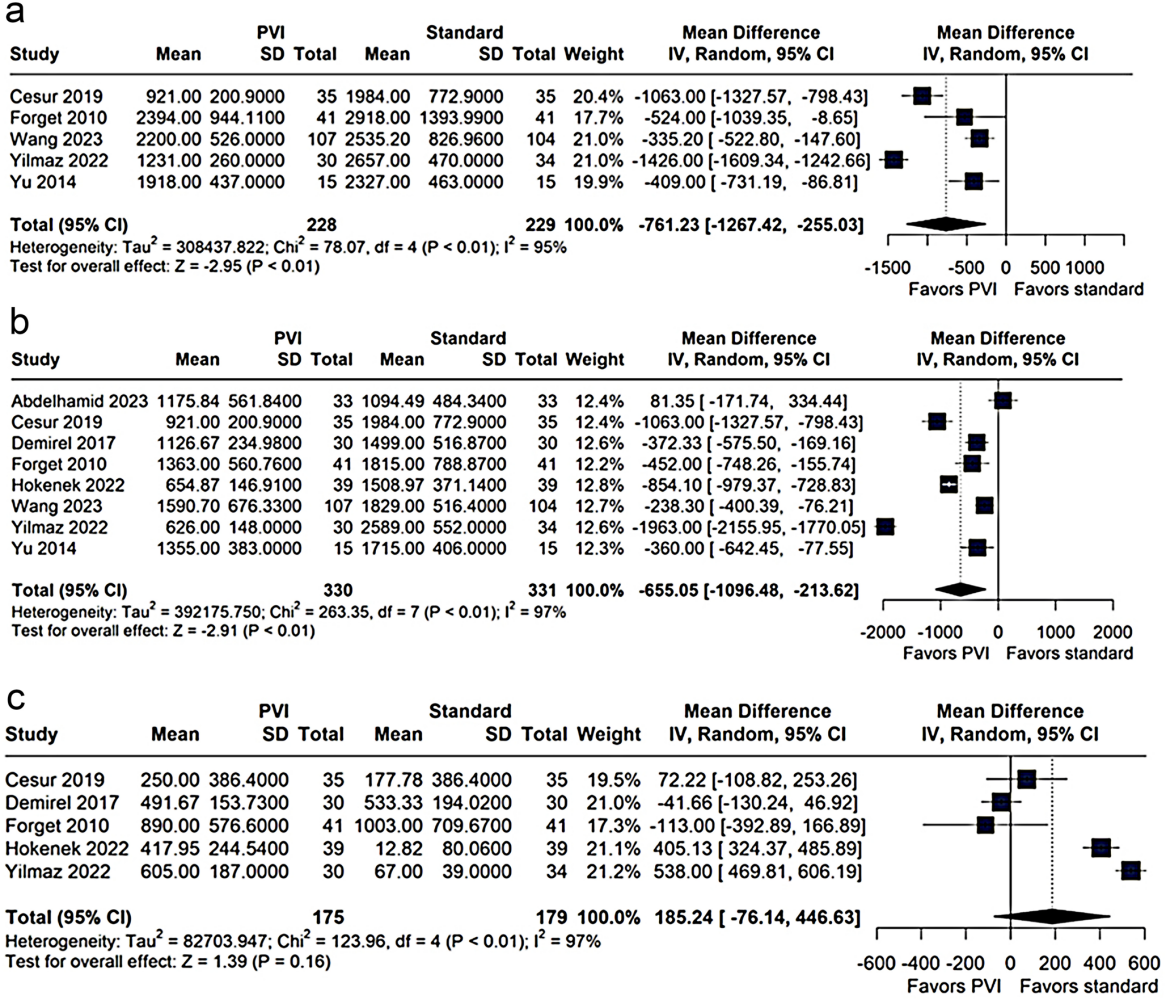
Meta-analysis comparing total fluid infused (**a**), total crystalloids infused (**b**), and total colloids infused (**c**) between the Pleth Variability Index (PVI) group and the standard management group. Mean differences (MD) and 95% confidence intervals (CI) were analyzed using a random-effects model

The pooled analysis indicated that the PVI-guided group received significantly less total fluid than the standard group (MD − 761.23 mL; 95% CI − 1267.42 to − 255.03; *p* < 0.01; I² = 95%, Fig. 2a). Notably, individual studies consistently showed a trend toward reduced fluid administration in the PVI group, with effect sizes ranging from − 1063.00 mL to − 335.20 mL.

#### Crystalloids and colloids infused

**Fig. 3:**
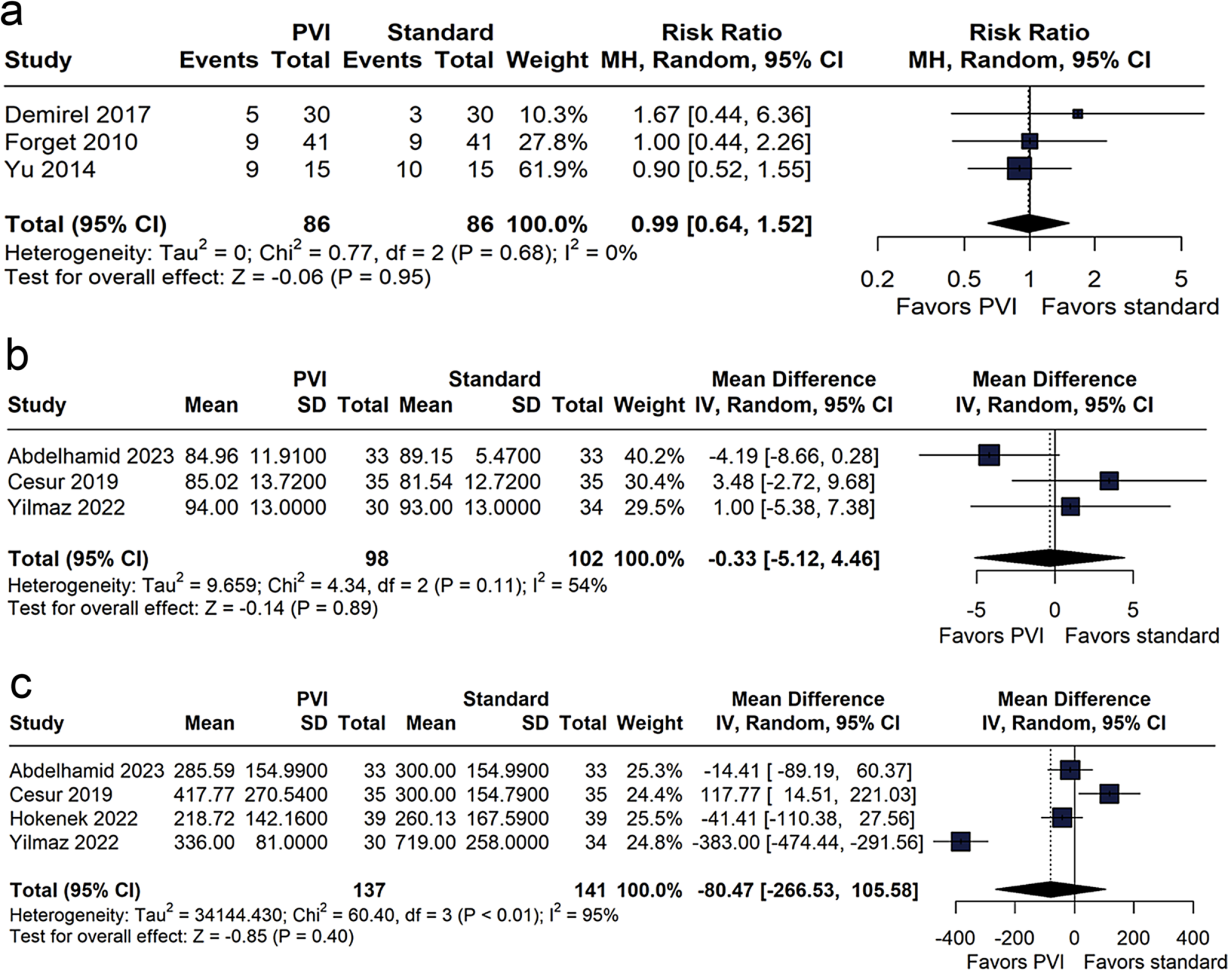
Meta-analysis evaluating the impact of the Pleth Variability Index (PVI) versus standard management on norepinephrine requirement (**a**), mean arterial blood pressure (**b**), and urine output (**c**). Results are presented as risk ratios (RR) or mean differences, with 95% confidence intervals (CI), using a random-effects model

Our results showed a significant reduction in the volume of crystalloids infused in the PVI-guided group compared with the standard group (MD − 655.05 mL; 95% CI − 1096.48 to − 213.62; *p* < 0.01; I² = 97%, Fig. 2b). In contrast, there were no significant differences between groups in colloid volume (MD 185.24 mL; 95% CI − 76.14 to 446.63; *p* = 0.16; I² = 97%, Fig. 3c).

Secondary Outcomes.

#### Norepinephrine requirement, MAP, and urine output

The proportion of patients requiring noradrenaline was comparable between the PVI-guided and standard care groups (RR 0.99; 95% CI 0.64 to 1.52; *p* = 0.95; I² = 0%, Fig. 3a). Likewise, no significant difference in MAP was observed between the groups (MD − 0.33 mmHg; 95% CI − 5.12 to 4.46; *p* = 0.89; I² = 54%, Fig. 3b). Analysis of urine output indicated no statistically significant difference between the groups (MD − 80.47 mL; 95% CI − 266.53 to 105.58; *p* = 0.40; I² = 95%, Fig. 3c).

Arterial pH, Bicarbonate, and Lactate.

**Fig. 4:**
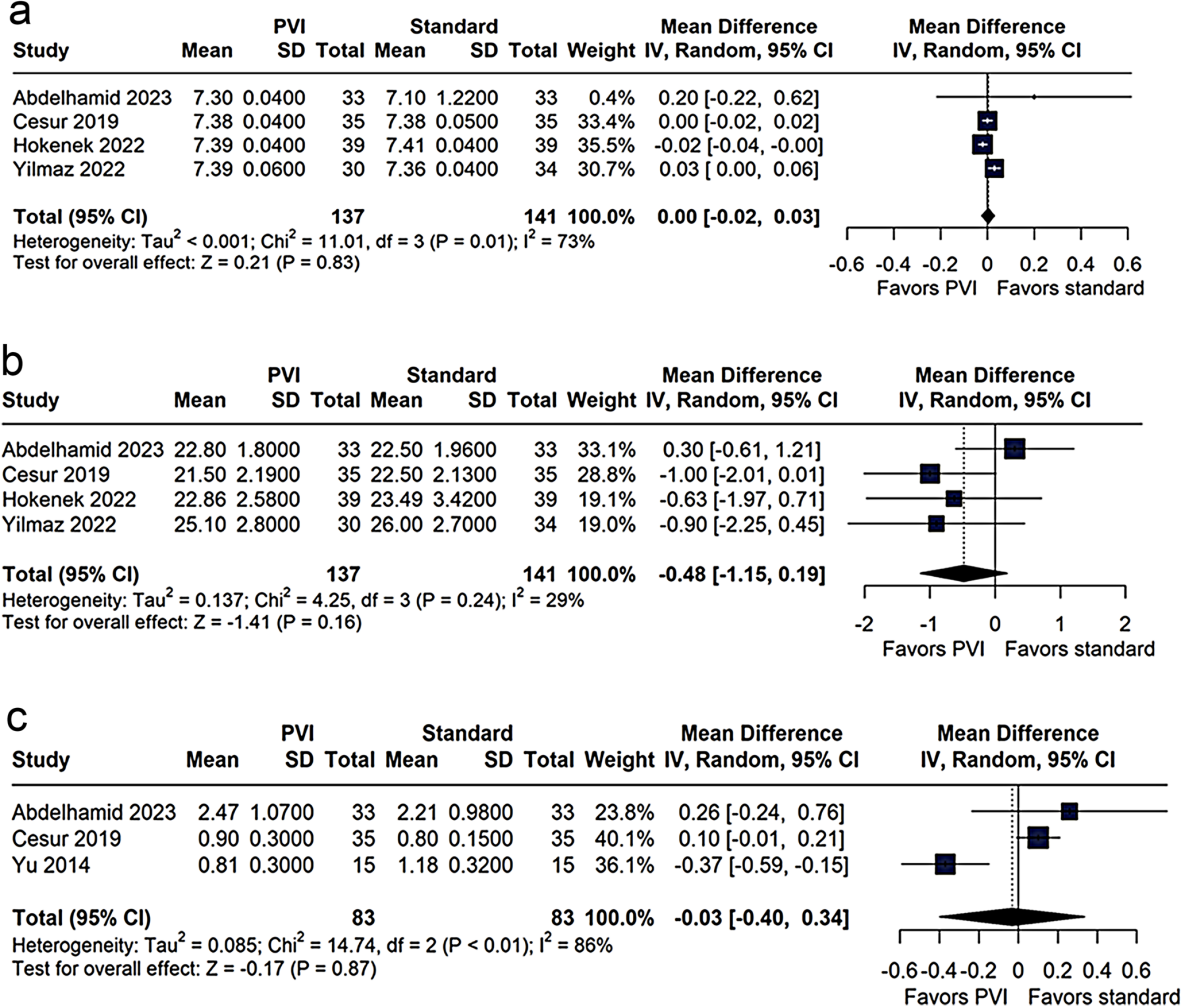
Meta-analysis comparing the effects of using the Pleth Variability Index (PVI) versus standard management on acid-base parameters (**a**), serum bicarbonate (**b**), and postoperative blood lactate (**c**). Mean differences and 95% confidence intervals (CI) were analyzed using a random-effects model

pH levels were comparable between groups (MD 0.00; 95% CI − 0.02 to 0.03; *p* = 0.83; I² = 73%, Fig. 4a). Bicarbonate levels differed by MD − 0.48 mmol/L (95% CI − 1.15 to 0.19; *p* = 0.16; I² = 29%, Fig. 4b), and intraoperative blood lactate levels were similarly matched (MD − 0.03 mmol/L; 95% CI − 0.40 to 0.34; *p* = 0.87; I² = 86%). One hour postoperatively, lactate levels showed no significant difference (MD 0.02 mmol/L; 95% CI − 0.14 to 0.18; *p* = 0.79; I² = 73%, Fig. 4c). Overall, no significant differences in acid-base parameters were observed between the groups.

#### Intraoperative blood loss, LOS, and postoperative creatinine

**Fig. 5:**
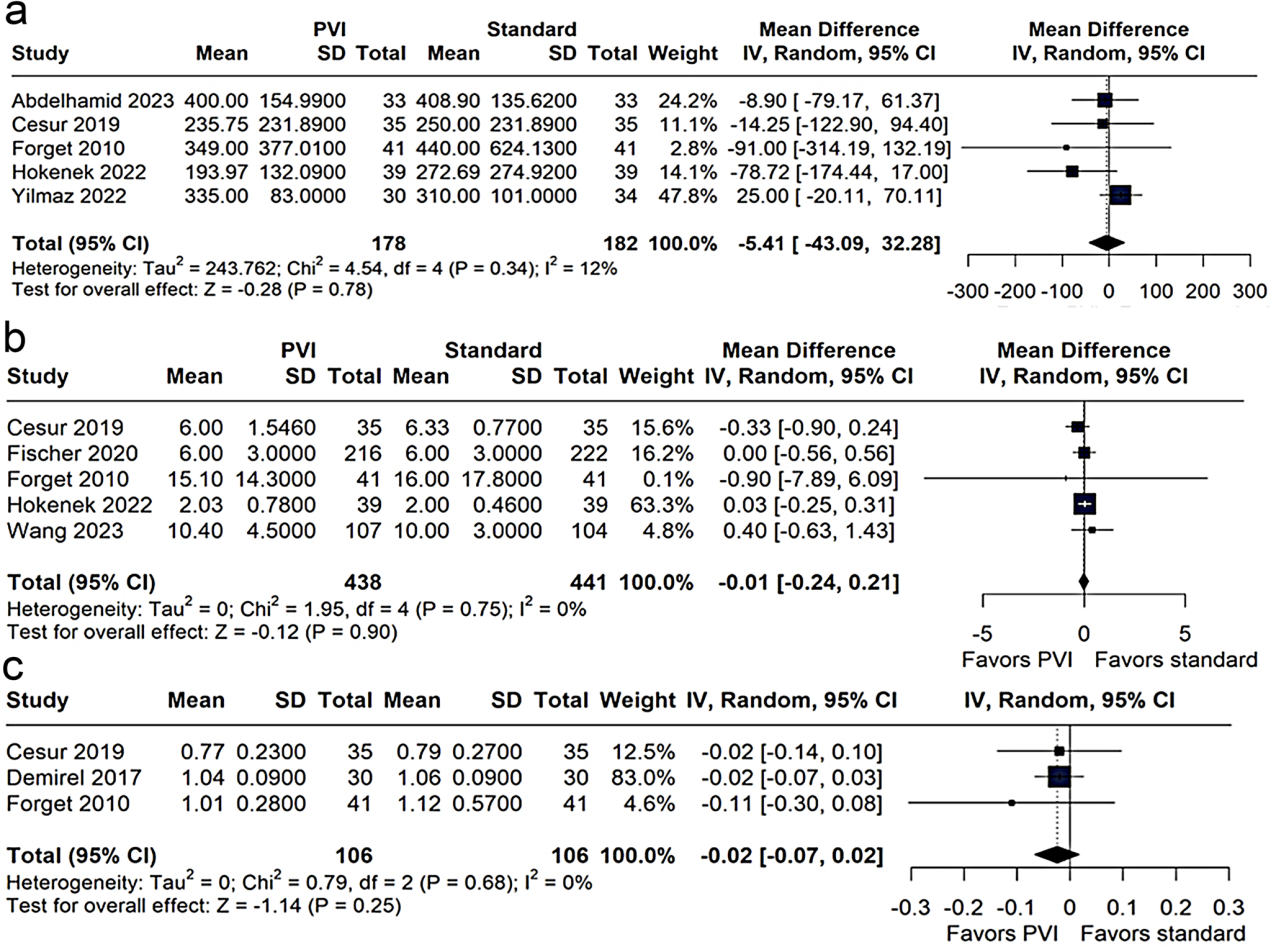
Meta-analysis evaluating blood loss (**a**), length of hospital stay (LOS) (**b**), and postoperative creatinine levels (**c**) in patients managed with the Pleth Variability Index (PVI) versus standard management. Results are presented as mean differences (MD) with 95% confidence intervals (CI) using a random-effects model

There was no statistical difference between groups regarding (MD − 5.41 mL; 95% CI − 43.09 to 32.28; *p* = 0.78; I² = 12%, Fig. 5a). The LOS was virtually the same (MD − 0.01 days; 95% CI − 0.24 to 0.21; *p* = 0.90; I² = 0%, Fig. 5b), and 24-hour postoperative creatinine levels also showed no significant difference (MD − 0.02 mg/dL; 95% CI − 0.07 to 0.02; *p* = 0.25; I² = 0%, Fig. 5c).

Subgroup analysis.

Abdominal surgeries.

**Fig. 6:**
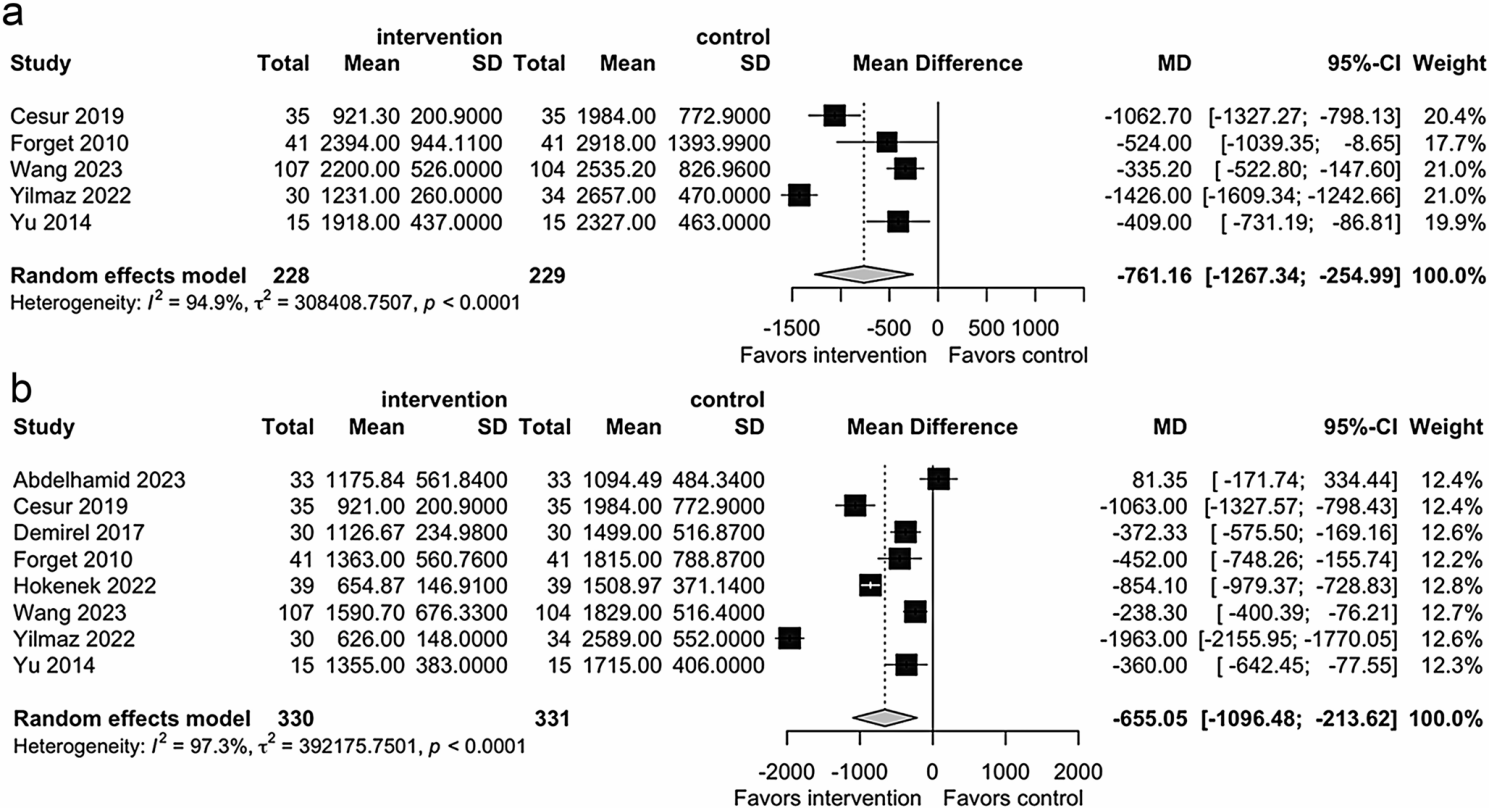
Subgroup analysis comparing total fluid infused (**a**) and total crystalloids infused (**b**) in abdominal surgeries between the intervention and control groups. Mean differences (MD) and 95% confidence intervals (CI) were analyzed using a random-effects model

In the subgroup analysis of abdominal surgeries, PVI-guided fluid management resulted in a significant reduction in crystalloid volume compared with conventional fluid management (MD − 655.05 mL; 95% CI − 1096.48 to − 213.62; *p* < 0.01; I² = 97.3%, Fig. 6a). A parallel reduction in total fluid volume was also observed (MD − 761.16 mL; 95% CI − 1267.34 to − 254.99; *p* < 0.01; I² = 94.9%, Fig. 6b), although high heterogeneity was noted in both outcomes, likely reflecting differences in surgical complexity and fluid management protocols. Sensitivity analyses confirmed these findings, suggesting that the reduction in total fluid volume was primarily driven by decreased crystalloid administration.

#### Sensitivity analyses

The leave-one-out sensitivity analysis confirmed the robustness of the PVI-guided intervention, as excluding any single study did not alter the direction or significance of the effect estimates. However, persistent high heterogeneity suggests that other factors (e.g., methodological differences or population characteristics) may contribute to the variability observed. The results for the sensitivity analysis are presented in Supplementary Figs. 1, 2.

#### Risk of bias assessment

Among the nine included studies, the majority were classified as having either a low risk of bias or some concerns. Specifically, eight out of nine studies were categorized as having an overall low risk of bias or some concerns in specific domains. Only one study was identified as having a high risk of bias [7] primarily due to concerns in the domain related to deviations from intended interventions (Supplementary Fig. 3).

The assessment showed that most studies had a low risk in key domains such as deviations from interventions, missing outcome data, and measurement of outcomes. However, concerns were noted in the randomization process for multiple studies, indicating potential limitations in allocation concealment or blinding methods.

There was no clear evidence of publication bias based on the visual inspection of the funnel plot for key outcomes (Supplementary Figs. 4 and 5). However, the small number of studies included in each individual analysis limits the ability to formally assess publication bias quantitatively. Some concerns in the risk of bias assessment.

## Discussion

This systematic review and meta-analysis of nine randomized controlled trials involving 1,105 surgical patients evaluated the effectiveness of PVI-guided goal-directed fluid management (PVI-GDFM) compared to conventional fluid management (CFM). Our findings demonstrate that PVI-GDFM significantly reduces total fluid and crystalloid administration without compromising hemodynamic stability, renal function, acid-base balance, or postoperative outcomes. These results support PVI as a viable, non-invasive tool for intraoperative fluid optimization, particularly in settings where invasive hemodynamic monitoring is impractical.

Among the primary outcomes, PVI-GDFM was associated with a significant reduction in total fluid volume (− 761.23 mL) and crystalloid administration (− 655.05 mL) compared to CFM. Despite this more restrictive approach, patients in the PVI group maintained stable hemodynamic parameters, including mean arterial pressure (MAP) and vasopressor requirements, with no significant differences relative to the control group. Renal function, assessed by intraoperative urine output and postoperative creatinine levels, was similarly preserved. Additionally, metabolic markers (arterial pH, bicarbonate levels, and blood lactate concentrations demonstrated no disruption in acid-base balance). Collectively, these findings suggest that reduced fluid administration under PVI guidance does not compromise tissue perfusion or organ function.

Subgroup analysis focusing on abdominal surgeries reinforced the clinical utility of PVI-guided fluid management in surgical contexts associated with substantial fluid shifts and increased risk of fluid overload. These procedures are often characterized by third-space losses, intra-abdominal pressure changes due to pneumoperitoneum, and surgical trauma, which make fluid balance particularly challenging. In this subgroup, PVI-guided therapy led to a more pronounced reduction in total fluid and crystalloid administration compared to conventional strategies, without inducing hemodynamic instability. Excessive fluid in these settings has been linked to impaired tissue healing, delayed gastrointestinal recovery, and cardiopulmonary complications [7, 8]. 

Our sensitivity analyses, including leave-one-out procedures, confirmed the robustness of these results and demonstrated that no single study disproportionately influenced the pooled estimates. Notably, Yu et al. [22] reported that PVI-guided therapy under combined general and epidural anesthesia significantly reduced fluid volumes and lactate levels, further supporting its effect on tissue perfusion.

While these results are promising, it is important to interpret them within the framework of modern perioperative care. In the context of enhanced recovery after surgery (ERAS) protocols, major improvements in clinical outcomes are increasingly driven by multimodal strategies rather than single interventions. Therefore, expecting a solitary change in fluid therapy to significantly affect outcomes such as mortality or hospital length of stay may be unrealistic. Instead, fluid optimization using tools like PVI should be viewed as a component that contributes incremental but meaningful gains in recovery when embedded in a comprehensive protocol. Although this analysis did not show a reduction in major postoperative complications, the ability to reduce intraoperative fluid volumes safely may still offer clinical value. PVI-GDFM can serve as a supportive, non-invasive tool for fluid optimization within multimodal perioperative care pathways, where cumulative effects, not individual interventions, drive outcome improvements.

We chose to conduct a subgroup analysis on abdominal surgeries due to their physiologic complexity and the availability of methodologically homogeneous data within this category. Other subtypes, such as orthopedic and gynecologic surgeries, were underrepresented and presented greater variability in design and endpoints, limiting the feasibility of separate subgroup analyses.

Although not part of our pooled analysis, kidney transplant recipients illustrate a compelling clinical scenario where non-invasive monitoring may be particularly beneficial. These patients often have fragile vascular access due to long-term dialysis or vascular disease, making the avoidance of unnecessary arterial or central venous cannulation crucial. In this context, continuous assessment of fluid responsiveness through PVI could provide valuable clinical information with minimal procedural risk. The transplant setting exemplifies a broader principle: in patients where invasive monitoring is contraindicated or impractical, PVI represents a practical alternative worthy of prospective investigation.

Looking forward, further research should prioritize high-quality, adequately powered randomized controlled trials that examine the long-term and patient-centered effects of PVI-guided fluid therapy. Standardization of PVI thresholds and fluid intervention protocols is also needed to reduce inter-study variability and enhance reproducibility. Comparative studies combining PVI with other dynamic monitors such as SVV or esophageal Doppler could clarify the best strategies for individualized perioperative care. Moreover, implementation studies assessing integration of PVI within ERAS protocols may provide insights into its cost-effectiveness and clinical scalability.

Despite the promising findings of this meta-analysis, several limitations warrant consideration. The high degree of heterogeneity in fluid volume outcomes likely reflects differences in surgical procedures, populations, and institutional practices. The absence of standardized PVI intervention thresholds across studies may have contributed to inconsistent effects. Nevertheless, subgroup and sensitivity analyses reinforced the direction and consistency of the findings. Additionally, the limited focus on long-term outcomes across the included trials restricts conclusions about broader postoperative benefits. We also acknowledge variability in CFM definitions; however, strict eligibility criteria and systematic risk-of-bias assessments helped ensure methodological rigor.

## Conclusion

This meta-analysis suggests that PVI-guided fluid management reduces intraoperative fluid administration without compromising physiological stability. These findings support the use of PVI as a non-invasive tool for individualized fluid optimization, particularly in abdominal surgeries and in patients where invasive monitoring is unfeasible. The heterogeneity observed highlights the need for further research to refine its application and define its role in contemporary perioperative medicine. 

## Electronic supplementary material

Below is the link to the electronic supplementary material.

Supplementary Material 1

## Data Availability

No datasets were generated or analysed during the current study.
