# Supplementary material for: Non-invasive goal-directed fluid therapy with the pleth variability index (PVI): a systematic review and meta-analysis
Source: J Clin Monit Comput. 2025 Aug 8;39(5):917–27. doi: 10.1007/s10877-025-01334-7 (PMC12474725; doi:10.1007/s10877-025-01334-7)

Supplemental Material:

Table of Contents

Supplementary Methods: Search strategy.

Supplementary Figure 1 - Leave one out sensitivity analysis: Total Fluid infused.

Supplementary Figure 2 - Leave one out sensitivity analysis: Total cristaloid infused.

Supplementary Figure 3 - Risk of Bias assessment (RoB-2).

Supplementary Figure 4 - Funnel plot: Total Fluids indufed.

Supplementary Figure 5 - Funnel plot: Total crystalloids infused.

Supplementary methods 1: Search strategy:

("Pleth Variability Index" OR “PVI” OR "Pulse Oximeter Variability" OR "Plethysmography Variability" OR "Non-Invasive Monitoring")

Supplementary Figure 1: Leave one out sensitivity analysis - Total Fluid infused:


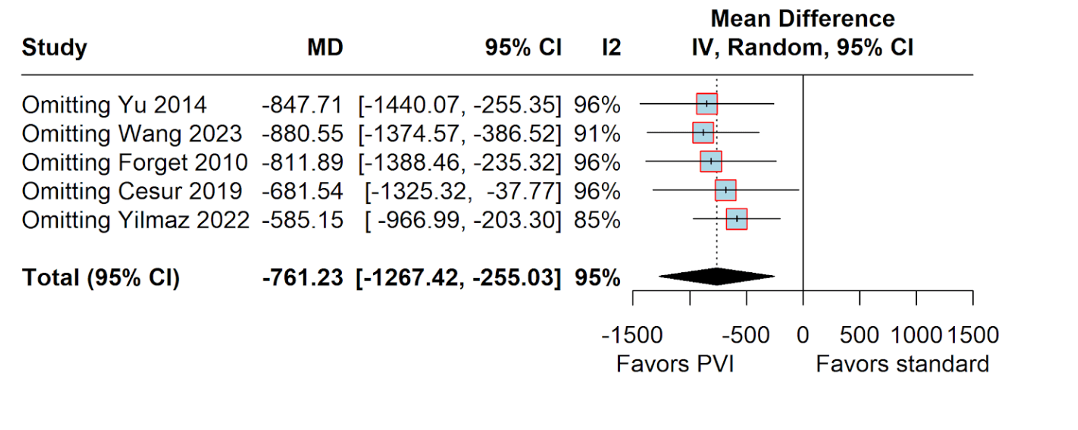


Supplementary Figure 2: Leave one out sensitivity analysis - Total crystalloid infused:


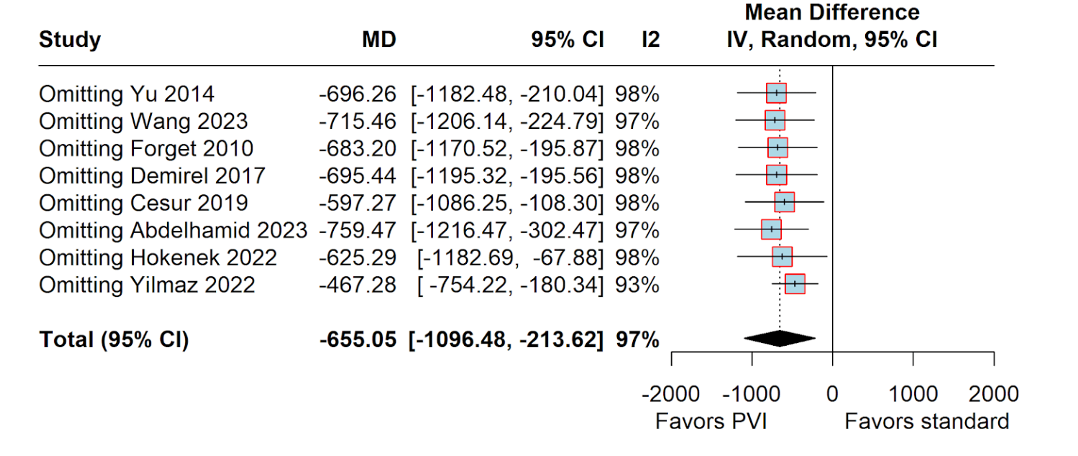


Supplementary Figure3: Risk of Bias assessment (RoB-2)


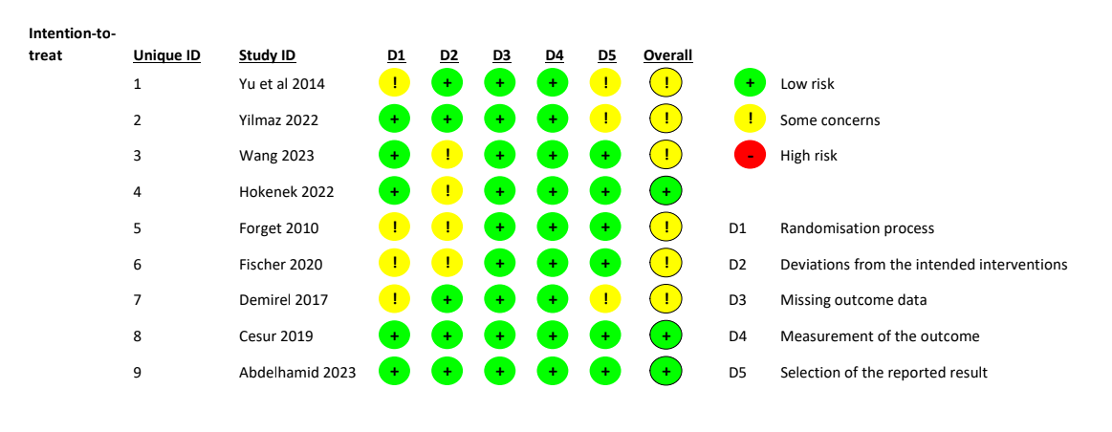


Supplementary Figure 4: Funnel plot: Total Fluids infused:


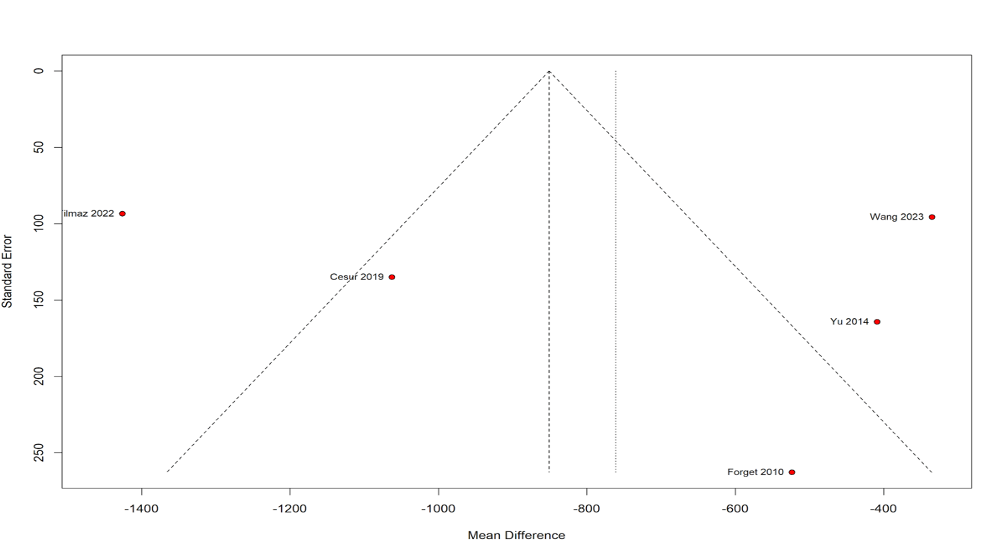


Supplementary Figure 5: Funnel plot: Total crystalloids infused:


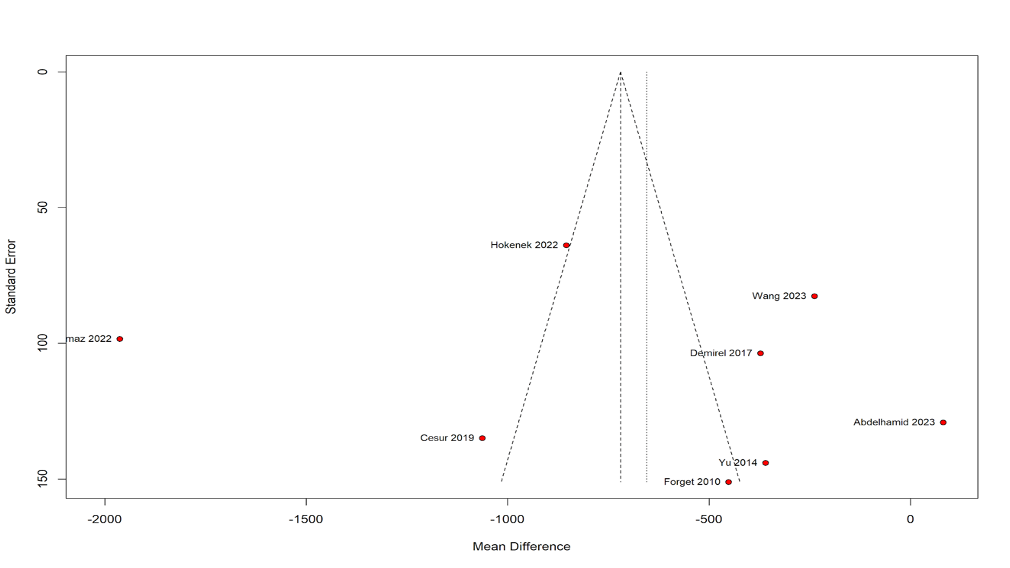

Supplement: Supplementary file 1 — Supplementary Material 1 [file 10877_2025_1334_MOESM1_ESM.docx]
